# 542 Mobile Burn Response Teams: A Scoping Review

**DOI:** 10.1093/jbcr/irac012.170

**Published:** 2022-03-23

**Authors:** Danielle H Fuchko, Kathryn King-Shier, Vincent Gabriel

**Affiliations:** University of Calgary, Calgary, Alberta; University of Calgary, Calgary, Alberta; University of Calgary, Calgary, Alberta

## Abstract

**Introduction:**

Advancements in the treatment of burns have reduced morbidity. However, the resources needed to deliver up-to-date care may be overwhelmed by mass casualty disasters. In 2021, the World Health Organization (WHO) recommended that countries prepare teams of deployable burn experts to assist with responding to a mass casualty disaster including burn patients. The aim of this scoping review was to identify existing literature regarding burn management mobile response team organization, describe the reported effectiveness of these teams, identify challenges in adopting the WHO recommendations and consider how the recommendations may be evolved.

**Methods:**

We conducted a scoping review of all literature types published up to February 2021. Searches of MEDLINE, EMBASE, Scopus, and CINAHL databases were conducted to identify reports informing or reporting the use of mobile burn care specialty teams that respond to events resulting in multiple burn-injured victims, including pediatric victims and military response to civilian events.

**Results:**

Of 5,737 identified reports, 24 publications were reviewed. Three distinct types of mobile burn response teams were identified: 1) teams organized by burn care networks; 2) government-organized medical disaster teams with burn-specific experts, and 3) the US Army Burn Flight Team. Teams have responded to events such as terrorist attacks by providing specialized burn supplies and personnel. Mobile burn response teams have demonstrated expert triage and stabilization advantages but are limited by the number of deployable specialists. A challenge in deploying a mobile team is the removal of experts from a burn centre.

**Conclusions:**

Although the WHO recommends increasing the number of mobile burn response teams available around the world, few countries have implemented this recommendation. A hybrid model where responders on scene communicate with burn centre experts to manage triage may address these challenges.

